# Concentration of mercury in human hair and associated factors in residents of the Gulf of Trieste (North-Eastern Italy)

**DOI:** 10.1007/s11356-022-23384-z

**Published:** 2022-10-21

**Authors:** Luca Cegolon, Elisa Petranich, Elena Pavoni, Federico Floreani, Nicolò Barago, Elisa Papassissa, Francesca Larese Filon, Stefano Covelli

**Affiliations:** 1 Occupational Medicine Unit, Department of Medical, Surgical & Health Sciences, University of Trieste, Trieste, Italy; 2 Public Health Department, University Health Agency Giuliano-Isontina (ASUGI), Trieste, Italy; 3 Department of Mathematics & Geosciences, University of Trieste, Trieste, Italy; 4 Department of Life Sciences, University of Trieste, Trieste, Italy

**Keywords:** Mercury exposure, Hair concentration, Fish consumption, Cinnabar, Marine contamination, Gulf of Trieste

## Abstract

The Gulf of Trieste (Northern Adriatic Sea, Italy) is the coastal area of the Mediterranean Sea most highly contaminated by mercury (Hg) due to fluvial inputs from the Isonzo/Soča River system, draining over 500 years’ worth of cinnabar extraction activity from the Idrija mining district (Western Slovenia). The aim of this research is to investigate the concentration of Hg in hair samples taken from the general population of the Friuli Venezia Giulia (FVG) Region coastal area, as a marker of chronic exposure to Hg. Three hundred and one individuals — 119 males and 182 females — were recruited by convenience sampling in Trieste in September 2021. An amount of approximately 100 mg of hair was collected from the occipital scalp of each participant to measure the respective Hg concentrations (expressed as mg/kg). Moreover, participants completed a self-report questionnaire collecting extensive socio-demographic and life-style information. A multiple linear regression analysis was employed to investigate factors associated with increased levels of Hg concentration in hair. A mean Hg concentration in hair of 1.63 mg/kg was found, slightly above the 1.0 mg/kg threshold recommended by the WHO for pregnant women and children, although still well below the no observed adverse effects level (NOAEL) of 10 mg/kg. Among respondents, 55.6% showed a Hg concentration in hair > 1 mg/kg, 22.9% > 2 mg/kg, and 2 participants exhibited Hg levels > 10 mg/kg. The adjusted mean hair Hg level increased in those subjects who reported a preference for shellfish/crayfish/mollusks (RC = 0.35; 95%CI: 0.16; 0.55), whereas it decreased in those who reported a preference for frozen fish (RC = -0.23; 95%CI: − 0.39; − 0.06). Though a risk alert for Hg exposure for coastal residents from FVG is deemed unnecessary at this time, it is recommended that pregnant women limit their ingestion of locally caught fish to < 4 servings/month.

**Supplementary Information:**

The online version contains supplementary material available at 10.1007/s11356-022-23384-z.

## Introduction

The health effects of mercury (Hg), a heavy metal in use for more than 3000 years, became an issue of global importance following the 1956 Minamata disaster in Japan, when methylmercury (CH_3_Hg^+^ or MeHg), the most toxic organic form of Hg, was released in the surrounding area for a considerable period of time, polluting the nearby sea and severely intoxicating more than 5000 local residents, some of whom eventually died (Ye et al. 2016). Another serious incident occurred in Iraq in 1971–1972, with 6500 cases of intoxication and 459 deaths following the ingestion of bread contaminated by MeHg as a result of a Hg-containing fungicide used to treat seed grain (Guzzi and La Porta 2008).

Methylmercury is a strong neurotoxin (Grandjean et al. 2010) and an endocrine disrupting chemical (Tan et al. 2009), highly toxic to the liver and the reproductive system of humans and other organisms (Crespo-López et al. 2009). Since it is highly soluble in lipids, MeHg is also detrimental to the central and peripheral nervous system (Rice et al. 2014).

Mercury is a global health concern, with dietary intake and occupational risks being the main exposure routes (Wang et al. 2021). The ingestion of fish is the dominant mode of exposure to MeHg for humans, which can bioaccumulate and biomagnify through the food chain in aquatic systems (NRC 2000).

After being ingested, MeHg is rapidly absorbed by red blood cells (RBC), bound to hemoglobin and distributed to various organs (Clarkson and Magos 2006; Guzzi and La Porta 2008). Elimination of Hg from the human body predominantly occurs via demethylation and fecal excretion of its inorganic form (Guzzi and La Porta 2008).

Since Hg tends to increase with fish intake (Castaño et al. 2015), its concentration in blood is used as an established marker of Hg exposure (Wilhelm et al. 2004; Castaño et al. 2019). Nevertheless, long-standing human exposure to Hg is typically assessed using urine and hair specimens (Airey 1983; WHO 2003; Barregard et al. 2006; Ye et al. 2016; Basu et al.^2018^). Whilst Hg in urine reflects the exposure to its inorganic form (IHg) originated from food-borne MeHg de-methylated in the human body (Castaño et al. 2015), MeHg increases its concentration in hair from blood flow by forming MeHg–cysteine complexes with the average hair-blood ratio in humans estimated to be approximately 250:1 μg/g-mg Hg/L (WHO 1990). However, in absence of acute exposure, Hg concentrations are much higher in hair than in blood (estimated ratios of 370:1) (Phelps et al. 1980; Shrestha and Fornerino 1982).

Although MeHg in the body combines with sulfur atoms of thiol ligands to form water soluble complexes, urinary excretion of MeHg is negligible (Guzzi and La Porta 2008), and it is considered a reliable qualitative biomarker for Hg exposure only when measuring the levels in hair is not possible (Esteban-López et al. 2022). Human hair is in fact the ideal biomarker of chronic exposure to MeHg (Thompson et al. 2014; Koenigsmark et al. 2021; Esteban-López et al. 2022) also taking into consideration the relatively low cost of sampling and the non-invasive nature of the procedure (Wang et al. 2021).

Once incorporated into the hair, Hg stabilises by irreversibly tying with sulfhydryl groups of cheratin, which constitutes up to 80–90% of hair, thus providing a continuous record of the duration of Hg exposure based on the typical rate of human hair growth of approximately 1 cm per month (Koenigsmark et al. 2021). Hg levels in hair reduce only with hair loss (Nielsen and Andersen 1991; Ye et al. 2016).

**Fig. 1:**
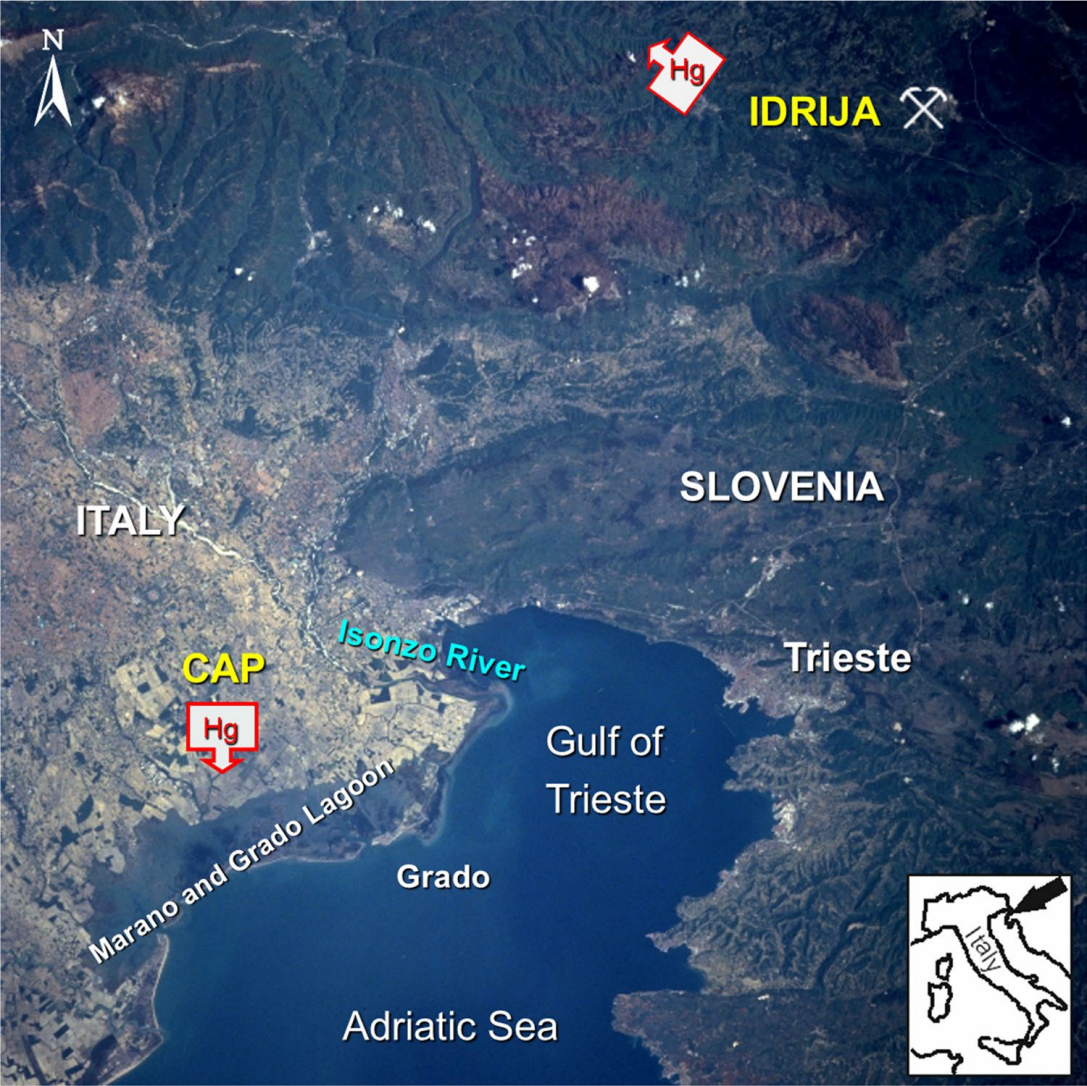
The Gulf of Trieste, where the participants, mainly residents, were recruited

The Gulf of Trieste (Northern Adriatic Sea, Italy) is the most highly Hg contaminated coastal area of the Mediterranean due to fluvial inputs from the Isonzo/Soča river system. Indeed, over 500 years of cinnabar (HgS) extraction activity from the Idrija mining district (western Slovenia) caused the contamination of water, soil, and sediments from the Isonzo/Soča River drainage basin (Horvat et al. 2002; Kotnik et al. 2005; Gosar and Žibret 2011; Kocman et al. 2011; Baptista-Salazar et al. 2017) as well as the marine-coastal environment (Horvat et al. 1999; Covelli et al. 2001) (Fig. 1). However, local contamination from the city of Trieste cannot be excluded since Hg in sediments was found in the old port area (Furlan et al. 1999) likely connected to urban sewage and local stream inputs which travel across the city and flows out into the port (Covelli et al. 2001).

The occurrence of Hg in the Gulf of Trieste has been investigated in several studies focused on coastal sediment contamination (Covelli et al. 2001, 2021), transport, and distribution of Hg associated with suspended particulate matter at the mouth of the Isonzo River (Covelli et al. 2006; 2007), as well as Hg cycling at the sediment–water interface (Emili et al. 2011; 2012). Nevertheless, few studies have investigated Hg concentrations in fish and shellfish in this coastal marine area (Kosta et al. 1978; Horvat et al. 1999).

Efficient trophic transfer of MeHg through aquatic food webs (biomagnification) results in Hg concentrations in predator species millions of times higher than those observed in surface water (Lavoie et al. 2013). More recently, interactions between Hg concentrations in seawater, sediments, plankton and the benthic rays — apex predators found in the Gulf of Trieste — were investigated as markers of Hg marine environmental contamination (Faganeli et al. 2018). The weight and age of benthic ray species were found to be correlated with Hg muscle concentration reaching up to 4.40 μg/g dw (Faganeli et al. 2018). However, it seems likely that efficient sedimentary MeHg demethylation and water column Hg(II) reduction are important factors preventing extensive contamination of marine biota in the Gulf of Trieste (Faganeli et al. 2014).

In view of the above, the aim of this study was to investigate Hg concentrations in human hair from volunteers recruited in Trieste, predominantly residents along the coast of the Friuli Venezia Giulia (FVG) Region.

## Methods

In this research study, 301 individuals — 119 males and 182 females — were recruited using convenience sampling from September 24 to 26, in 2021, at an outdoor gazebo located in the main square (Piazza Unità d’Italia) of Trieste, the capital city of the FVG Region. Participants were recruited during the celebration of the 10th anniversary of “*Trieste Next*” Festival of Scientific Research, a long-standing event dedicated to research dissemination.

All participants gave their written informed consent after reading a form and receiving an explanation regarding the objectives of the study.

An amount of approximately 100 mg of hair was collected with stainless steel scissors from the occipital scalp of each participant. Hair samples were then labeled and stored separately in individual polyethylene bags until analysis.

All participants also filled out a self-administered questionnaire which took approximately 5 min to complete, containing extensive information on the socio-demographic profile of respondents and their lifestyles and general habits, especially related to diet and fish consumption (Table S1).

In addition to participation are of residence and participation to in hobbies and free time activities which may increase the risk of Hg exposure, the questionnaire also included questions on smoking, alcohol consumption, number of dental amalgams, bruxism, nasal dyspnea, consumption of chewing gum, dietary supplements, and use of protective creams, contact lenses use and history of kidney disease.

Furthermore, the questionnaire delved into water intake (amount consumed per day), source of drinking water (mineral, aqueduct, filtered aqueduct, well, filtered well), number of meals containing fish eaten per week, type (fresh, frozen, canned) and size of fish preferred. Three main categories of fish were considered: large fish (swordfish, tuna, cod); small/medium size (anchovies, sardines, herring, sea bass, mullet, flounder, bream, eel), and shellfish/crayfish/mollusks (squid, crab, shrimp, clam, octopus, mussel, lobster, crayfish).

Total Hg in hair samples was measured using a Direct Mercury Analyzer (DMA-80, Milestone, Sorisole, Italy), according to the EPA Method 7473 (USEPA 1998). The limit of detection (LOD) was equal to 0.004 mg/kg, calculated multiplying the standard deviation coming from the average of 10 blanks by three and dividing the result obtained by the slope of the calibration curve. The accuracy of the method for the analytical determination of Hg was verified by analyzing a certified reference material (CRM) for a better representation of the results:

- ERM-DB001 (0.365 ± 0.028 mg/kg; Human Hair CRM, European Reference Materials).
- Acceptable recoveries were obtained, ranging between 98 and 106%.

### Statistical analysis

Statistical descriptive analysis was performed, calculating frequencies, percentages, mean, standard deviation, median, and inter-quartile range. Since Hg in hair was not normally distributed (Figure S1a), a log-transformation of the outcome measure was performed (Figure S2b).

A univariate linear regression analysis was employed to investigate the association of each factor with the outcome (log-transformed Hg concentration). A backward stepwise selection procedure was used to fit a multivariable linear regression model from explanatory factors displayed in Table 1 (excluding water intake, due to a high number of missing values).

**Table 1:** Frequency distribution of factors and univariable linear regression analysis (ordinary vs. log-transformed outcome). Number (N), column percentages (%), regression coefficients  unadjusted (RC) and adjusted (aRC) with 95% confidence intervals with 95% confidence intervals. Mean (M) ± standard deviation (SD); IQR: interquartile range. M: missing information

Variables		Strata		*N* (Col %)	Univariable linear regression analysis	
					Ordinary outcome RC (95%CI)	Outcome log-transformed aRC (95%CI)
Mercury concentration in hair (mg/kg) (M: 1)		Median (IQR)		1.20 (0.78; 1.86)		
		M ± SD		1.63 ± 1.50		
		< 1.00		113 (37.5)		
		1.01–2.00		119 (39.5)		
		2.01–10.00		67 (22.3)		
		> 10.00		2 (0.7)		
Sex (M: 1)		Females		180 (59.8)	Reference	Reference
		Males		121 (40.2)	0.37 (0.03; 0.71)	0.16 (0.00; 0.32)
Age (years) (M: 3)		Median (IQR)		29.5 (21; 49)		
		M ± SD		34.7 ± 18.8	0.01 (− 0.00; 0.02)	0.01 (0.00; 0.02)
Domicile (M: 11)		Inland/off Trieste gulf		11 (3.8)	Reference	Reference
		Coastal (off Trieste gulf)		69 (23.8)	1.24 (0.28; 2.19)	0.38 (− 0.07; 0.83)
		Trieste		210 (72.4)	0.25 (− 0.15; 0.66)	0.13 (− 0.06; 0.33)
Residence area (M: 3)		Industrial		12 (4.0)	0.20 (− 0.68; 1.08)	− 0.12 (− 0.53; 0.30)
		Urban		220 (73.6)	− 0.21 (− 0.62; 0.20)	− 0.06 (− 0.25; 0.13)
		Rural		67 (22.4)	Reference	Reference
BMI (kg/m^2^) (M: 5)		Median (IQR)		22 (20; 24.5)		
		M ± SD		22.5 ± 4.9		
		< 25		222 (75.0)	Reference	Reference
		25–29		58 (19.6)	0.04 (− 0.39; 0.48)	0.12 (− 0.08; 0.33)
		30 +		16 (5.4)	0.14 (− 0.63; 0.90)	0.07 (− 0.29; 0.43)
Nasal dyspnea (M: 4)		No		238 (79.3)	Reference	Reference
		Yes		60 (20.1)	0.03 (− 0.40; 0.45)	0.06 (− 0.14; 0.26)
Seniority of work (M: 126)		Median (IQR)		168 (48; 324)		
		M ± SD		199.4 ± 168.7		
		< 48		42 (24.0)	Reference	Reference
		48–155		43 (24.6)	− 0–11 (− 0.71; 0.50)	0.05 (− 0.25; 0.35)
		156–299		38 (21.7)	0.05 (− 0.58: 0.67)	0.17 (− 0.14; 0.48)
		300 +		52 (29.7)	0.36 (− 0.22; 0.95)	0.15 (− 0.14; 0.44)
Hobbies at risk for Hg exposure		No		287 (95.4)	Reference	Reference
		Yes		14 (4.6)	− 0.02 (− 0.82; 0.78)	0.04 (− 0.34; 0.41)
Water intake (liters/day) (M: 136)		Median (IQR)		1.5 (1; 2)		
		M ± SD		1.6 ± 0.6		
		< 1		63 (34.1)	0.05 (− 0.43; 0.53)	0.07 (− 0.17; 0.30)
		1–2		50 (27.0)	− 0.23 (− 0.75; 0.30)	− 0.05 (− 0.30; 0.20)
		2 +		72 (38.9)	Reference	Reference
Water source (M: 136)		Mineral		41 (24.9)	Reference	Reference
		Aqueduct		113 (68.5)	− 0.06 (− 0.60; 0.47)	− 0.02 (− 0.27; 0.23)
		Aqueduct (filtered)		9 (5.5)	0.27 (− 0.81; 1.35)	0.17 (− 0.34; 0.67)
		Well		1 (0.6)	− 0.27 (− 3.25; 2.70)	0.09 (− 1.30; 1.47)
		Well (filtered)		1 (0.6)	− 0.22 (− 3.20; 2.75)	0.13 (− 1.26; 1.51)
Smoking habit (N. cigarettes/day) (M: 4)		Non (or ex) smoker		264 (88.6)	Reference	Reference
		< 15		31 (10.4)	0.04 (− 0.52; 0.60)	− 0.01 (− 0.27; 0.25)
		15 +		3 (1.0)	− 0.68 (− 2.40; 1.04)	− 0.36 (− 1.16; 0.45)
Wine intake (liters/day) (M: 6)		0		183 (61.8)	Reference	Reference
		≤ 0.5		105 (35.5)	0.16 (− 0.21; 0.53)	0.13 (− 0.04; 0.31)
		> 0.5		8 (2.7)	0.23 (− 0.85; 1.30)	0.30 (− 0.20; 0.80)
Chewing gum consumption (M: 6)		No		257 (86.8)	Reference	Reference
		Yes		39 (13.2)	− 0.09 (− 0 − 60; 0.41)	− 0.03 (− 0.27; 0.20)
Bruxism (M: 11)		No		234 (80.4)	Reference	Reference
		Yes		57 (19.6)	0.03 (− 0.41; 0.47)	− 0.00 (− 0.20; 0.21)
Supplements (M: 5)		No		221 (74.4)	Reference	Reference
		Yes		76 (25.6)	0.08 (− 0.31; 0.47)	0.09 (− 0.09; 0.28)
History of kidney disease (M: 5)		No		287 (96.6)	Reference	Reference
		Yes		10 (3.4)	− 0.08 (− 1.03; 0.87)	0.11 (− 0.34; 0.55)
Protective skin creams (M: 4)		No		133 (44.8)	Reference	Reference
		Yes		164 (55.2)	0.03 (− 0.32; 0.37)	0.08 (− 0.08; 0.24)
Dental filling amalgams (*N*)		0		216 (71.8)	Reference	Reference
		1–3		43 (14.0)	0.10 (− 0.39; 0.59)	0.15 (− 0.08; 0.38)
		4 +		42 (14.0)	0.13 (− 0.37; 0.62)	0.13 (− 0.10; 0.36)
Amalgams fillings removed/placed in the past 2 months (M: 12)		No		262 (90.7)	Reference	Reference
		Yes		27 (9.3)	− 0.15 (− 0.75; 0.45)	− 0.10 (− 0.38; 0.18)
Contact lenses (M: 5)		No		250 (84.5)	Reference	Reference
		Yes		46 (15.5)	− 0.01 (− 0.49; 0.46)	0.05 (− 0.17; 0.28)
Fish intake (N. meals/months) (M: 6)		< 4		116 (39.3)	Reference	Reference
		4		113 (38.3)	0.26 (− 0.13; 0.64)	**0.19 (0.01; 0.37)**
		> 4		66 (22.4)	**0.64 (0.19; 1.09)**	**0.28 (0.07; 0.49)**
Preferred fish provenance		Fresh	No	131 (43.5)	Reference	Reference
			Yes	170 (56.5)	0.34 (− 0.00; 0.68)	0.15 (− 0.01; 0.31)
		Frozen	No	196 (65.1)	Reference	Reference
			Yes	105 (34.9)	* − 0.23 (− 0.40; − 0.05)*	* − 0.10 (− 0.18; − 0.01)*
		Canned	No	242 (80.4)	Reference	Reference
			Yes	59 (19.6)	− 0.32 (− 0.75; 0.10)	− 0.13 (− 0.33; 0.07)
Fish size intake	Big *	No		37 (12.4)	Reference	Reference
		Yes		261 (87.6)	− 0.11 (− 0.62; 0.41)	− 0.03 (− 0.27; 0.21)
	Small/medium**	No		73 (24.5)	Reference	Reference
		Yes		225 (75.5)	**0.65 (0.26; 1.03)**	**0.35 (0.17; 0.53)**
	Shell/crayfish/mollusks’ ^$^	No		62 (20.8)	Reference	Reference
		Yes		236 (79.2)	**0.44 (0.02; 0.86)**	**0.34 (0.15; 0.53)**

^*^Sword fish, tuna, cod

^**^Anchovies, sardines, sea bass, sea bream, ribon, gilt-head bream, grey mullet, mullet, plaice, conger

^$^Scampi, prawn, shrimp, sea cicada, crab, lobster, sea crayfish, clams, mussel, squids, calamary, octopus

Bold: risk factor; Italics: protective factor

Results of linear regression were expressed as adjusted regression coefficients (aRC) with a 95% confidence interval (CI).

Missing data were excluded, and a complete case analysis was performed. The Stata 14.2 software (Stata Corporation, College Station, TX, USA) was used for the analysis.

## Results

Three hundred and one individuals agreed to participate in this study. The majority of participants were females (59.8%=180/301), with a median age of 30 years (IQR: 21–49; range: 3–79) (Table 1).

Most participants (70.2%) reported living along the coast of the Gulf of Trieste — including the municipalities of Trieste, Sistiana, Duino Aurisina, Monfalcone, and Grado — whereas 23.8% were residents in coastal sites off the Gulf. Two hundred and twenty individuals (73.6%) reported living in an urban area, whereas 22.4% (=67/299) were residents in a rural area and only 4% (=12/299) in an industrial area or nearby (Table 1).

Overweight individuals (BMI > 30 kg/m^2^) accounted for 5.4% (=16/296) of the total number of participants, whereas 75% (=222/296) of the studied population exhibited a BMI < 25 kg/m^2^. Respondents suffering from nasal dyspnea were 20.1% (= 60/297) and 4.6% (= 14 /301) had hobbies posing a risk of Hg exposure. Water consumption was rather balanced, as 34.1% (=63/185) of participants reported drinking < 1 L/day, 27.0% (=50/185) between 1 and 2 L/day, and 38.9% (=72/185) > 2 L/day. The majority of the study population drank tap water (68.5% = 113/165), whereas only 24.9% (= 41/165) consumed mineral water (Table 1).

Most individuals, 88.6% (=264/298) were non-smokers or ex-smokers whereas 10.4% (=31/298) and 1.0% (=3/298) reported smoking < and > 15 cigarettes per day, respectively. The majority of respondents (61.8% = 183/296) reported that they did not drink wine, and 35.5% (= 105/296) drank < 0.5 L of wine per day. Thirty-nine out of 295 (13.2%) respondents consumed chewing gum, and 19.6% (=57/291) suffered from bruxism. Seventy-six out of 296 (25.6%) respondents used dietary supplements, 55.4% (= 164 / 297) used protective skin creams, 15.5% (= 46/296) were contact lenses users, and only 3.4% (= 10/296) reported being affected by any type of kidney disease (Table 1).

Two hundred and sixteen participants (71.8%) had no dental amalgams; 14.3% (=43/301) had 4 amalgams and 14.0% (=42/301) > 4 amalgams. Twenty-seven (9.3%) of the participants reported having undergone dental procedures involving insertion or removal of dental amalgams in the previous 2 months (Table 1).

**Fig. 2:**
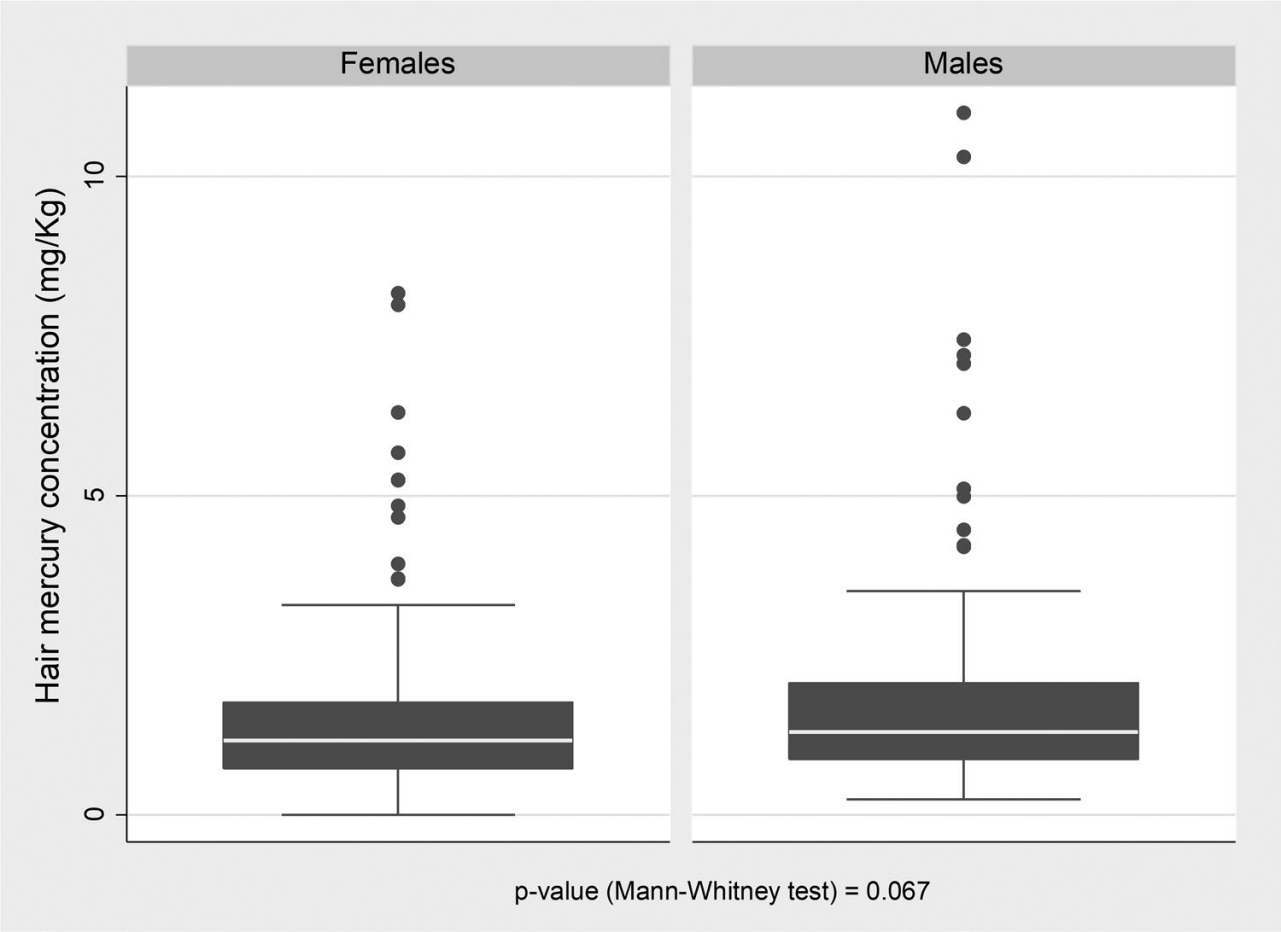
Box plot displaying the distribution of Hg concentration in hair (mg/kg) by sex

Figure 2 shows the box plots for the distribution of Hg concentration in hair by sex of respondents. The median and mean values for Hg hair concentration in males were 1.29 mg/kg (IQR: 0.87; 2.06) and 1.85 ± 1.79 mg/kg respectively, whereas in females they were 1.16 mg/kg (IQR: 0.72; 1.75) and 1.48 ± 1.23 mg/kg respectively. As can be seen in Table 1, at a univariate analysis Hg concentration in hair slightly increased with age (RC = 0.01; 95% CI: 0.00; 0.02).

**Fig. 3:**
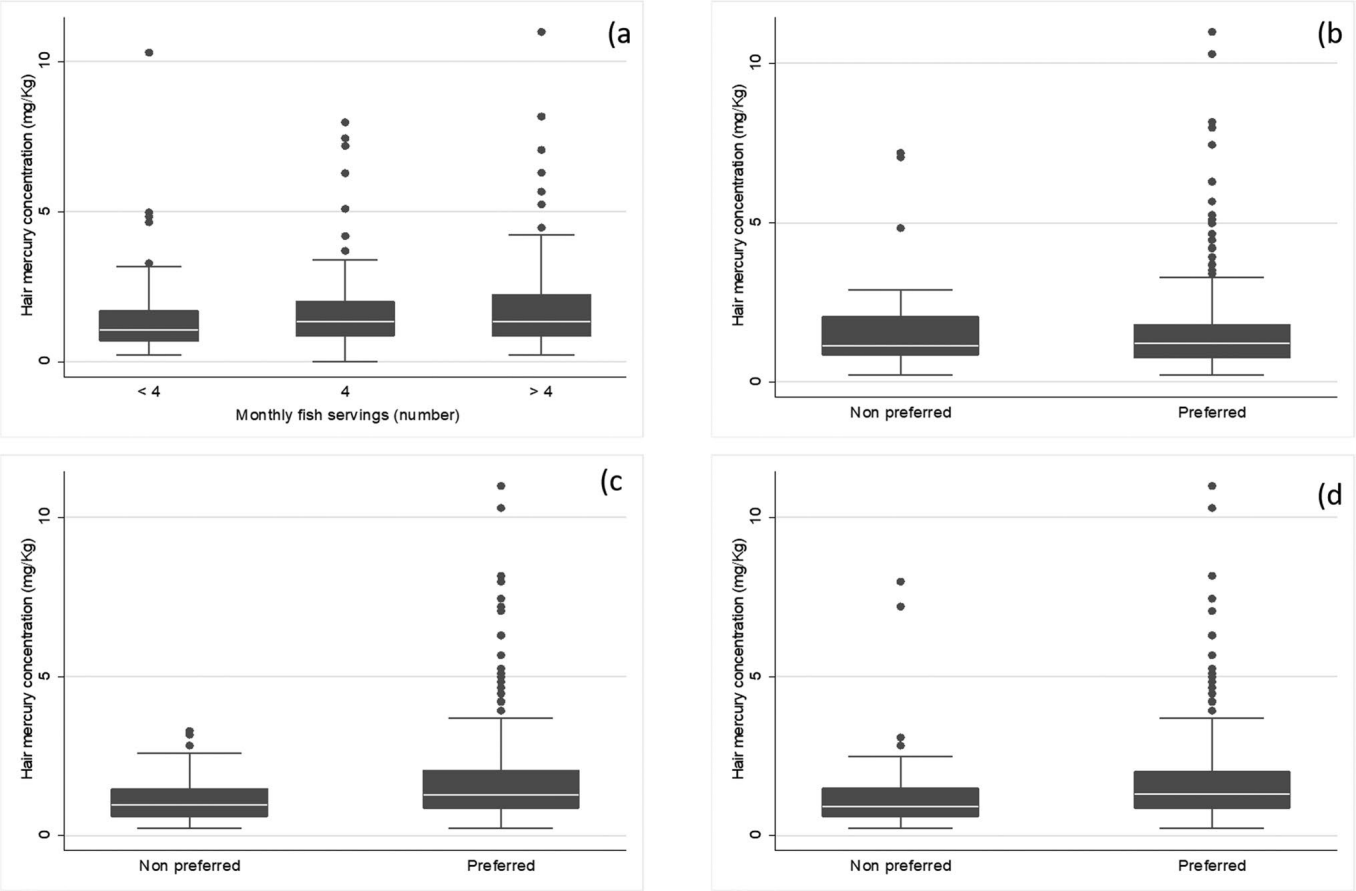
**a** Distribution of Hg concentration in hair (mg/kg) by number of monthly fish servings; **b** distribution of Hg concentration in hair (mg/kg) by preferred consumption of large-sized fish (No vs. yes); **c** distribution of Hg concentration in hair (mg/kg) by preferred consumption of small/medium sized fish (No vs. yes); **d** distribution of Hg concentration in hair (mg/kg) by preferred consumption of shellfish/cray fish/mollusks (Noyes vs. yesno)

Fish consumption was evenly distributed, as 39.3% (=116/295) participants reported eating fish < 4 times a month, 38.3% (=113/295) at least 4 times a month and 22.4% (=66/295) > 4 times a month. Fresh fish was preferred by 56.5% (= 170/301) respondents, followed by frozen (34.9%=105/301) and canned fish (19.6%= 59/301). The distribution of the type of fish consumed was also rather balanced, as (87.6%=261/298) respondents reported eating large fishes, (75.0%=225/298) small-medium size fishes, and (79.2%= 236/298) shellfish/crayfish/mollusks (Table 2). Figure 3a–d shows the box plots for Hg distribution in hair by fish consumption (monthly meals and type of fish preferred).

**Table 2:** Distribution of mercury concentration in hair by fish intake. Number (*N*); column percentages (%); mean ± standard deviation (SD)

			Monthly fish intake (N. meals)			Type of fish consumed					
			< 4	4	> 4	Big size		Small/medium size		Shell/cray fish/mollusks	
						No	Yes	No	Yes	No	Yes
Mercury concentration in hair (mg/kg)	Mean ± SD		1.40 ± 1.24	1.65± 1.36	2.04 ± 2.00	1.74 ± 1.58	1.63 ± 1.486	1.15 ± 0.70	1.80 ± 1.64	1.29 ± 1.34	1.73 ± 1.52
	Median (IQR)		1.081 (0.70; 1.70)	1.34 (0.87; 2.00)	1.34 (0.86; 2.24)	1.15 (0.86; 2.05)	1.21 (0.78; 1.81)	0.98 (0.62; 1.47)	1.28 (0.87; 2.05)	0.91 (0.61; 1.49)	1.30 (0.87; 2.00)
	STRATA	*N* (%)	*N* (%)	*N* (%)	*N* (%)	*N* (%)	*N* (%)	*N* (%)	*N* (%)	*N* (%)	*N* (%)
	**≤ 1.00**	113 (37.5)	51 (44.0)	37 (32.7)	21 (31.8)	13 (35.1)	98 (37.8)	37 (50.7)	74 (32.9)	33 (53.2)	78 (33.1)
	**1.01–2.00**	119 (39.5)	43 (37.1)	48 (42.5)	27 (40.9)	14 (37.8)	104 (39.9)	26 (35.6)	92 (40.9)	19 (30.7)	99 (42.0)
	**2.01–10.00**	67 (22.3)	21 (18.1)	28 (24.8)	17 (25.8)	10 (27.0)	57 (21.8)	10 (13.7)	57 (25.3)	10 (16.4)	57 (24.2)
	**> 10.00**	2 (0.7)	1 (0.9)	0	1 (1.5)	0	2 (0.8)	0	2 (0.9)	0	2 (0.9)

The median Hg concentration in hair was 1.2 mg/kg (IQR: 0.78; 1.86), with a mean of 1.63 ± 1.50 mg/kg (Table 1). One hundred and thirteen participants (37.5%) exhibited Hg hair concentration < 1.1 mg/kg, 39.5% (=119/301) between 1.1 and 2.1 mg/kg, 22.3% (=67/301) within the range 2.1–10 mg/kg, and only 2 people (0.7%) exhibited hair Hg concentrations > 10.1 mg/kg.

Table 2 shows the mean distribution of Hg concentration in hair by size and monthly consumption of fish. As can be noted, almost 80% of participants exhibited Hg levels < 2 mg/kg, and Hg concentrations increased with the amount of fish eaten per month and the ingestion of small/medium size fish or shellfish/crayfish/mollusks.

Participants who reported consuming fish < 4 times monthly revealed a mean concentration of Hg in hair equal to 1.40 ± 1.24 mg/kg and a median of 1.08 (IQR: 0.70; 1.70) mg/kg. Those who reported eating fish 4 times per month exhibited 1.65 ± 1.36 mg/kg mean Hg levels in hair, with a median of 1.34 (IQR = 0.87; 2.00) mg/kg. The respective estimates for participants consuming > 4 monthly fish servings increased to 2.04 ± 2.00 mg/kg and 1.34 (IQR: 0.86; 2.24) mg/kg (Table 2).

Table 3 displays the results of multivariable linear regression analysis. A preference for the consumption of shellfish/crayfish/mollusks was significantly associated with higher mean levels of Hg concentration in hair (RC = 0.35; 95%CI: 0.16; 0.55). In contrast, the preferred ingestion of frozen fish was correlated to a decreased mean level of Hg concentration in hair samples (RC =  − 0.23; 95%CI: − 0.39; − 0.06).

**Table 3:** Multivariable linear regression (outcome measure log-transformed). Regression coefficients (RC) with 95% confidence interval (95%CI). Model fitted on 296 complete (case analysis) observations

Factors	Strata	RC (95%CI)
Frozen fish preferred	No	Reference
	Yes	− 0.23 (− 0.39; − 0.06)
Shell/crayfish/molluscs	No	Reference
	Yes	0.35 (0.16; 0.55)
Residence	Industrial	− 0.10 (− 0.52; 0.32)
	Urban	0.07 (− 0.12; 0.26)
	Rural	Reference

Figure S2 shows that the distribution of residuals for log-transformed Hg was approximately linear.

## Discussion

The mean Hg concentration in hair (1.63 ± 1.50 mg/kg) found in this study exceeded the 1.0 mg/kg threshold recommended by the WHO for pregnant women and children, although it was still well below the no observed adverse effects level (NOAEL) of 10 mg/kg (UNEP 2008).

Since fish from contaminated aquatic, marine, or freshwater systems is the main source of MeHg exposure in the general population, WHO recommends levels of 1 mg/kg and 0.5 mg/kg wet weight (w/w) for predatory and non-predatory fish, respectively (WHO 2007). In Europe, the current Hg limits in food items is 0.5 mg/kg, with an exception of 1 mg/kg for top predators (e.g., tuna, swordfish, and related species, EC 1881/2006) and other benthic species.

Whilst the US Food and Drug Administration (FDA 2022) estimates that most people have a daily food-related Hg exposure of 50 ng/kg, a dose not thought to be harmful to humans (Aprea et al. 2021; FDA 2022), high Hg (605 ± 210 ng/g) as well as MeHg (147 ± 37 ng/g ww) contents were found in Manila clams from the Marano and Grado Lagoon, which is adjacent to the Gulf of Trieste (Giani et al. 2012). The Lagoon is also subject to Hg contamination, primarily due to suspended sediment particles originating from the Soča/Isonzo River drainage basin (Covelli et al. 2007). In addition, Hg was used as a catalyst in a chlor-alkali plant (CAP) and discharged in an unregulated manner into the Aussa-Corno River system from 1949 to 1984, after which cleaner technologies were reportedly adopted (Piani et al. 2005). This river system flows to the central sector of the Lagoon where it is estimated that approximately 186 t of Hg of industrial origin have been deposited. Previous studies revealed high Hg levels in sediments (Acquavita et al. 2012), as well as evidence of bioaccumulation of metal along the trophic chain (Brambati 2001). Since almost all the participants in the present survey were residents of Trieste and the surrounding areas, it can be argued that the majority of the fresh fish and shellfish purchased and consumed were from the Adriatic Sea. However, we cannot exclude that fresh products may also reach Trieste and the neighboring coastal areas from the above lagoon environment.

Marine (fresh, canned, or frozen) fish is considered a more relevant source of Hg exposure than freshwater fish (Morrissette et al. 2004). Despite large fish being predators occupying the top-level of the aquatic trophic network, featuring the highest Hg content following biomagnification (De Almeida Rodrigues et al. 2019), the lack of association between Hg levels in hair samples and the consumption of large fish (swordfish, tuna, cod) in the present study is likely due to the ban on tuna fishing in the Gulf of Trieste. Indeed, edible tuna is imported to FVG Region from foreign countries, and ingestion of local fish is limited to other fish species. Nonetheless, overseas provenance of the other two fish categories shall not be ruled out. The significantly lower Hg levels in hair in relation to the consumption of frozen fish may be attributable to the overseas provenance and/or fish processing since a substantial reduction in bioaccessible Hg fractions in fish has been observed after cooking compared to raw fillets (Costa et al. 2022).

### Mercury exposure and fish consumption

Mercury concentration in scalp hair varies as a function of geographical areas and fish intake, both in Italy and elsewhere (Okati and Esmaili-sari 2018, Kirichuck et al. 2020). For instance, among 606 pregnant women delivering in Trieste during the period from 2007 to 2009, the mean concentration of hair Hg was lower (1.06 mg/kg) than the present study and only moderately correlated with fish intake (Valent et al. 2013b). Likewise, in another study conducted on residents of Naples (Campania region, Southern Italy) including both sexes, 115 females vs. 122 males, the mean Hg concentration in hair was 0.6 mg/kg, ranging between 0.22 and 3.40 mg/kg (Diez et al. 2008).

In a larger sample of 224 residents from 3 municipalities from the same bay (Eastern Sicily), the pooled median Hg content in hair was 1.47 mg/kg, 1.90 mg/kg in Augusta, 1.24 mg/kg in Melilli and 1.00 mg/kg in Priolo (Bonsignore et al. 2016). Again, increasing concentrations of Hg from both blood and hair specimens were detected among respondents who reported ingesting higher quantities of locally caught fish. In particular, the highest value of Hg in blood (33.6 μg/L) was found in a subject eating locally caught fish 3–4 times/week, whereas a respondent who reported seldom ingesting locally caught seafood revealed the lowest blood concentration of Hg (0.10 μg/L) (Bonsignore 2016).

A further study found a mean hair Hg concentration of 6.45 ± 7.03 mg/kg among 96 fishermen without dental problems aged 35–45 years sampled from 6 different coastal areas of Sicily (Italy), significantly higher (*p* < 0.001) than 96 controls not employed in the maritime sector (Giangrosso et al. 2016).

In summary, the concentrations of Hg in hair found in the present study were comparable to those found in residents of Sicily (Bonsignore 2016) but higher than those found for residents of Naples (Diez et al. 2008) and lower than those found in Sicilian fishermen (Giangrosso et al. 2016), suggesting that in high risk occupational categories or geographical areas Hg levels should be closely monitored.

### Other risk factors for mercury exposure

In line with other studies, the Hg concentration in hair found in this study was not influenced by age (Szynkowska and Pawlaczyk 2007; Karabedian et al. 2009; Michalak et al. 2014; Kirichuk et al. 2020; Munir et al. 2021). However, this is in contrast to other studies which report a correlation between increasing levels of Hg concentration in hair and age (Shah et al. 2016; Esteban-López et al. 2022), particularly in males (Wyatt et al. 2017).

Amalgam dental fillings are considered a potential source of IHg exposure for the human organism (Factor-Litvak et al. 2003; Bates 2006), since elemental Hg vapour (Hg^0^) may be released as a result of mastication (Sallsten et al. 1996), nocturnal bruxism (Isacsson et al. 1997), or teeth whitening products (Robertello et al. 1999) and subsequently oxidized into inorganic divalent Hg (Hg^2+^) and then absorbed (Clarkson 1997).

Nonetheless, hair Hg concentration was not influenced by number of dental fillings amalgams or amalgam fillings removed/replaced in the past 2 months. Whilst the latter finding is consistent with some other studies (Diez et al. 2008; Esteban-López et al. 2022), an investigation of 60 children reported higher Hg levels in urine those with amalgam fillings (Levy et al. 2004).

### Mercury exposure and human health

Neuro-developmental effects have reportedly been associated with in utero exposure and a maternal hair Hg concentration of 1.0 mg/kg or higher (NRC 2000). Consequently, the WHO recommends mean Hg concentrations of < 1.0 mg/kg in scalp hair of pregnant women and children (WHO 2007, 2008) and the US Environmental Protection Agency (EPA) fixed a reference daily exposure of 0.1 μg/kg for MeHg (USEPA 2001, 2010, 2021).

A prospective multi-center cohort study (NAC-II) is ongoing in four Adriatic countries (Italy, Slovenia, Croatia, and Greece) to investigate the association between prenatal Hg exposure from maternal fish consumption and childhood neuro-development (Valent et al. 2013b). In a single center study from the latter cohort focusing on 606 children and mother dyads delivering at the Institute for Maternal & Child Health in Trieste from 2007 to 2009 and residing in the coastal area of the FVG region, children’s cognitive development at 18 months of age increased with fish intake and the intelligence quotient of the respective mothers (Valent et al. 2013a, b), but not with pre-natal Hg exposure, regardless the specimen analyzed (hair, blood, or umbilical cord). A subsequent pooled analysis from the above multi-center NAC-II cohort, examining 1308 mother–child pairs, reported weak yet partially inconsistent evidence of an inverse relationship between Hg maternal concentrations (0.70 mg/kg in maternal hair; 2.4 ng/g in maternal blood; 3.6 ng/g in cord blood; 0.6 ng/g in breast milk) and the motor score of their children at 18 months, with cognitive and language functions not being affected (Barbone et al. 2019). An update follow-up study at 7 years of age is ongoing in the Gulf of Trieste to clarify whether low levels of Hg exposure may still have a detrimental effect on children’s neurodevelopment (Brumatti et al. 2021).

### Strengths and limitations

In line with most open literature on this topic, this study also relies on a convenience sampling strategy, which could be a potential source of selection bias influencing the generalisability of the findings. Nonetheless, this study fills a gap in the literature by providing evidence of environmental exposure to mercury among the general population of a coastal area that has been historically contaminated by this metal. Furthermore, the study population is relatively large as compared to other published studies.

## Conclusions

In the present study, higher mean concentrations of Hg in hair were found in subjects who reported a preference for consuming shellfish/crayfish/mollusks, largely fished in the Gulf of Trieste.

Whilst the mean levels of Hg in hair (1.63 mg/kg) detected in the present study sample are not alarming, a great proportion (56%) of participants showed concentrations of Hg higher than the threshold exposure recommended by the WHO for scalp hair in pregnant women and children (1 mg/kg). The evidence on health effect of newborns in relation to low dose maternal exposure to Hg in coastal areas of the FVG Region is still inconclusive. Furthermore, a debate is ongoing as to whether the beneficial effects of selenium (Se) from fish could offset the detrimental effect of Hg in children’s neurodevelopment, also considering its antagonist effect against Hg. Moreover, the evaluation of the effect of fish consumption on human health should also take into account the different ratio of Se/Hg concentration by fish species.

In conclusion, although a risk alert for Hg exposure seems inappropriate for coastal residents of the FVG Region, it appears prudent for pregnant women and children to limit the ingestion of local fish to < 4 servings/month.

Further studies are recommended on larger samples, using a stronger study design, also collecting information Hg levels in blood and cross-checking Hg exposure with follow-up health data.

## Supplementary Information

Below is the link to the electronic supplementary material.

Supplementary file1 (DOC 87 KB)

Supplementary file2 (DOC 71 KB)

## Data Availability

The datasets generated during and/or analyzed during the current study are available from the corresponding author on reasonable request.
