# Correction to: Concentration of mercury in human hair and associated factors in residents of the Gulf of Trieste (North‑Eastern Italy)

**DOI:** 10.1007/s11356-022-24098-y

**Published:** 2022-11-10

**Authors:** Luca Cegolon, Elisa Petranich, Elena Pavoni, Federico Floreani, Nicolò Barago, Elisa Papassissa, Francesca Larese Filon, Stefano Covelli

**Affiliations:** 1 Occupational Medicine Unit, Department of Medical, Surgical & Health Sciences, University of Trieste, Trieste, Italy; 2 Public Health Department, University Health Agency Giuliano-Isontina (ASUGI), Trieste, Italy; 3 Department of Mathematics & Geosciences, University of Trieste, Trieste, Italy; 4 Department of Life Sciences, University of Trieste, Trieste, Italy

**Correction to: Environmental Science and Pollution Research**

10.1007/s11356-022-23384-z

The correct Table 2 is shown in this paper.

**Table 2:** Distribution of mercury concentration in human hair by fish intake

			Monthly fish intake (Number of meals)			Type of fish consumed					
			<4	4	>4	Big size		Small/medium size		Shell/cray-fish/mollusks	
						No	Yes	No	Yes	No	Yes
Mercury concentration in hair (mg/kg)	Mean ± SD		1.40 ± 1.24	1.65 ± 1.36	2.04 ± 2.00	1.74 ± 1.58	1.63 ± 1.48	1.15 ± 0.70	1.80 ± 1.64	1.29 ± 1.34	1.73 ± 1.52
	Median (IQR)		1.08 (0.70; 1.70)	1.34 (0.87; 2.00)	1.34 (0.86; 2.24)	1.15 (0.86; 2.05)	1.21 (0.78; 1.81)	0.98 (0.62; 1.47)	1.28 (0.87; 2.05)	0.91 (0.61; 1.49)	1.30 (0.87; 2.00)
	**STRATA**	**N (%)**	**N (%)**	**N (%)**	**N (%)**	**N (%)**	**N (%)**	**N (%)**	**N (%)**	**N (%)**	**N (%)**
	**≤ 1.00**	113 (37.5)	51 (44.0)	37 (32.7)	21 (31.8)	13 (35.1)	98 (37.8)	37 (50.7)	74 (32.9)	33 (53.2)	78 (33.1)
	**1.01-2.00**	119 (39.5)	43 (37.1)	48 (42.5)	27 (40.9)	14 (37.8)	104 (39.9)	26 (35.6)	92 (40.9)	19 (30.7)	99 (42.0)
	**2.01-10.00**	67 (22.3)	21 (18.1)	28 (24.8)	17 (25.8)	10 (27.0)	57 (21.8)	10 (13.7)	57 (25.3)	10 (16.4)	57 (24.2)
	**>10.00**	2 (0.7)	1 (0.9)	0	1 (1.5)	0	2 (0.8)	0	2 (0.9)	0	2 (0.9)

Number (N); column percentages (%); Mean ± standard deviation (SD); median with Interquartile range (IQR)

