# Supplementary material for: Concentration of mercury in human hair and associated factors in residents of the Gulf of Trieste (North-Eastern Italy)
Source: Environ Sci Pollut Res Int. 2022 Oct 21;30(8):21425–37. doi: 10.1007/s11356-022-23384-z (PMC9938062; doi:10.1007/s11356-022-23384-z)

Figure S1. **a)** Distribution of mercury concentration in hair (mg/kg) by sex; **b)** Log-transformed mercury concentration in hair.


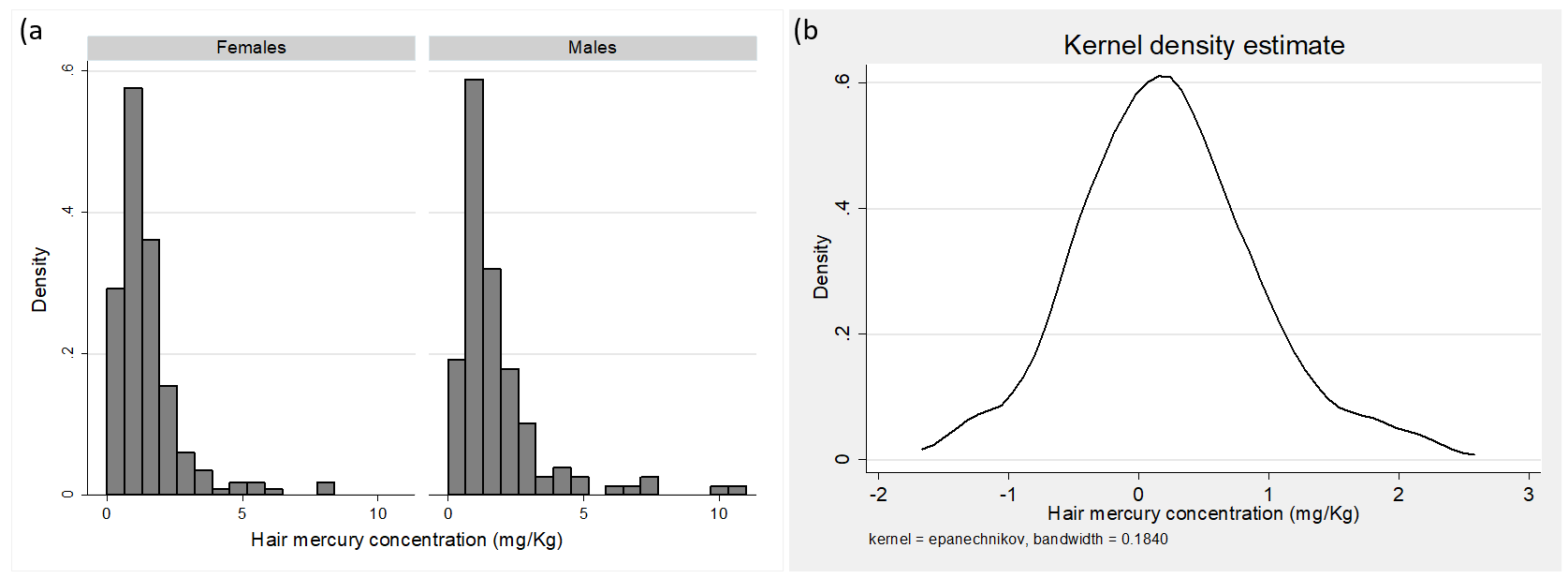


Figure S2. Distribution of residuals of log-transformed mercury concentrations in hair (mg/kg).


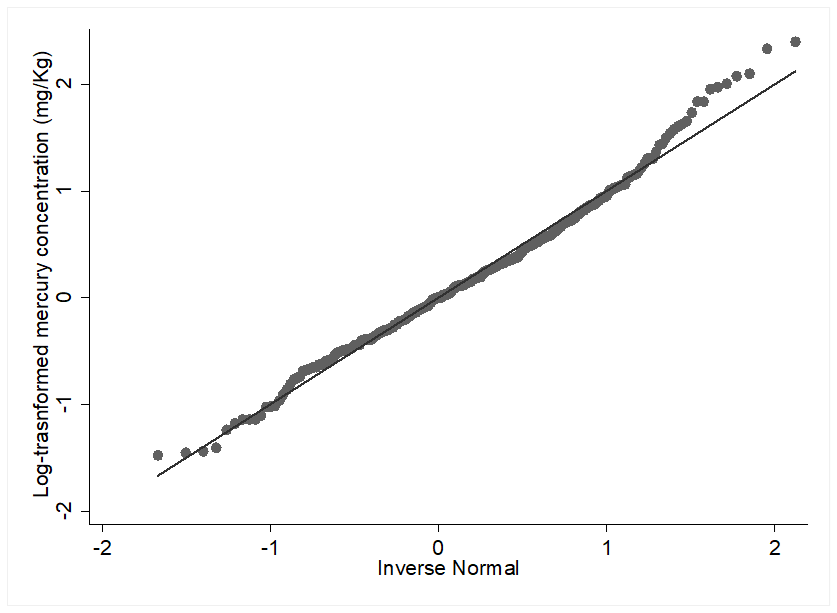

Supplement: Supplementary file 1 — Supplementary file1 (DOC 87 KB) [file 11356_2022_23384_MOESM1_ESM.doc]
