# Supplementary material for: Concentration of mercury in human hair and associated factors in residents of the Gulf of Trieste (North-Eastern Italy)
Source: Environ Sci Pollut Res Int. 2022 Oct 21;30(8):21425–37. doi: 10.1007/s11356-022-23384-z (PMC9938062; doi:10.1007/s11356-022-23384-z)
Supplement: Supplementary file 2 — Supplementary file2 (DOC 71 KB) [file 11356_2022_23384_MOESM2_ESM.doc]

Table S1. Questionnaire containing extensive information on the socio-demographic profiles of respondents and their lifestyles and habits.

# Survey Questionnaire on Mercury Exposure

| **Personal data** | Name | |  | | | | | |
| --- | --- | --- | --- | --- | --- | --- | --- | --- |
|  | Family Name | |  | | | | | |
|  | DOB | |  | | | | | |
|  | Address | |  | | | | | |
|  | e-mail | |  | | | | | |
|  | Sex | | - Female | | | - Male | | |
|  | Weight (Kg) | |  | | |  | | |
|  | Height (mt) | |  | | |  | | |
| **Occupation** | …………………………………… since  N. months/year ………….. | | ……………………………………….. since  N. months/year ………….. | | | ……………………………………….. since  N. months/year ………….. | | |
| **Residence area** | - Industrial   (< 500 mt from an industrial area) | | - Urban   (> 500 mt from an industrial area) | | | - Rural   (with limited road traffic and low population density) | | |
| **Type of industrial activity near your home** |  | | | | | | | |
| **Risky Hobbies for mercury exposure** |  | | | | | | | |
| **Number of dental amalgamsi** |  | | | | | | | |
| **Insertion/removal of dental amalgams in the past 2 months** | - Yes | | - No | | |  | | |
| **Nasal dyspnoea** | - Yes | | - No | | |  | | |
| **Bruxism** | - Yes | | - No | | |  | | |
| **Usual chewing-gum consumption** | - Yes | | - No | | |  | | |
| **Type (mineral, aqueduct, well) and daily amount of water intake** |  | | | | | | | |
| **Wine** | - No | | - ≤ ½ liter/day | | | - > ½ liter/day | | |
| **Smoking** | - No   (or smoking quitted > 1 year ago) | | - ≤ 15 cigarettes/day   (or pipe or sigar) | | | - > 15 cigarettes/day | | |
| **Number of fish meals** | - 1/ month | - 2 /month | | - 3 /month | - 1 /week | | - 2 /week | - > 2 /week |
| **Preferred fish** | - Fresh | | - Frozen | | | - Canned | | |
| **Fish type preferred** | Fish   - Sword fosh - tuna - cod - sardine - anchovy - sea bass - sea bream - ribon - gilt-head bream - grey mullet - mullet - plaice - conger | | Shellfish/crayfish   - scampi - prawn - shrimp - sea cicada - crab - lobster - sea crayfish | | | Molluscs   - clam - mussel - squid - calamary - octopus | | |
| **Use of Skin creams** | - Yes | | - No | | |  | | |
| **Use of contanct lenses** | - Yes | | - No | | |  | | |
| **Use of omeopathic drugs**  **or supplements** | - Yes | | - No | | |  | | |
| **Pre-existing/past renal diseases** | - Yes | | - No | | |  | | |

I …………………………………………………………………. herewith authorize the use of my personal data for this approved scientific study aiming to measure mercury concentration in human hair, in compliance with European General Data Protection Regulation (GDPR).

Date Signature

………………………………… ………………………………………………………………..
